# From Angiotensin IV to Small Peptidemimetics Inhibiting Insulin-Regulated Aminopeptidase

**DOI:** 10.3389/fphar.2020.590855

**Published:** 2020-10-15

**Authors:** Mathias Hallberg, Mats Larhed

**Affiliations:** ^1^ The Beijer Laboratory, Division of Biological Research on Drug Dependence, Department of Pharmaceutical Biosciences, BMC, Uppsala University, Uppsala, Sweden; ^2^ Department of Medicinal Chemistry, Science for Life Laboratory, BMC, Uppsala University, Uppsala, Sweden

**Keywords:** angiotensin IV, peptidemimetics, aminopeptidase N, cystinyl aminopeptidase, insulin-regulated aminopeptidase

## Abstract

It was reported three decades ago that intracerebroventricular injection of angiotensin IV (Ang IV, Val-Tyr-Ile-His-Pro-Phe) improved memory and learning in the rat. There are several explanations for these positive effects of the hexapeptide and related analogues on cognition available in the literature. In 2001, it was proposed that the insulin-regulated aminopeptidase (IRAP) is a main target for Ang IV and that Ang IV serves as an inhibitor of the enzyme. The focus of this review is the efforts to stepwise transform the hexapeptide into more drug-like Ang IV peptidemimetics serving as IRAP inhibitors. Moreover, the discovery of IRAP inhibitors by virtual and substance library screening and direct design applying knowledge of the structure of IRAP and of related enzymes is briefly presented.

## Introduction

**Figure 1:**
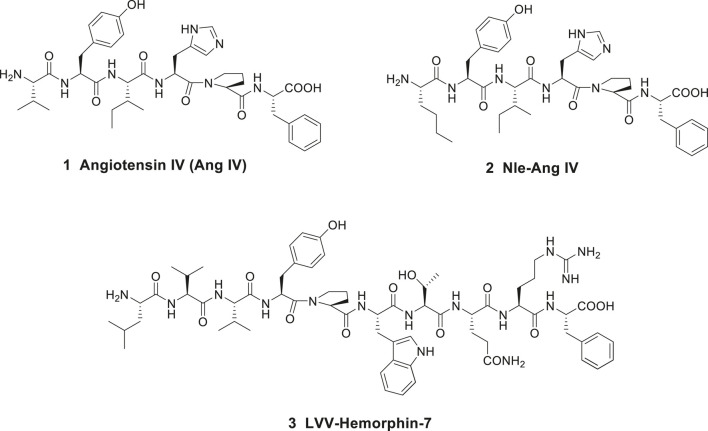
Peptides with high affinity to the angiotensin IV (AT4) receptor.

Angiotensin IV (Ang IV) is a small bioactive peptide in the renin-angiotensin system (RAS) formed after proteolytic degradation of angiotensin II (Ang II). In 1988, Braszko et al. (1988), at the Medical University of Bialystok, Poland demonstrated that intracerebroventricular injection of Ang IV (Val-Tyr-Ile-His-Pro-Phe), **1** in rat (1 nmol dose) improved memory and learning (Figure 1). Furthermore, it was reported that Ang IV affects both passive and conditioned avoidance response as well as motor activity. The impact of the hexapeptide in various experimental models, e.g., for Barnes maze, swim mazes as well as radial arm mazes were subsequently explored (Wright et al., 1993; Wright et al., 1996; Wright et al., 1999; Lee et al., 2003; Braszko et al., 2008; De Bundel et al., 2009). Related Ang IV analogues were studied as well, such as Nle-Ang IV **2** (Nle-Tyr-Ile-His-Pro-Phe) and the endogenous LVV-hemorphin-7 **3** (Leu-Val-Val-Tyr-Pro-Trp-Thr-Gln-Arg-Phe), both with structural similarities to Ang IV at the N-terminal part of the peptides (Figure 1). Nle-Ang IV **2** and LVV-hemorphin-7 **3**, the latter formed after degradation of β-globin, were demonstrated to be strong promoters of memory retention and retrieval in rats (Lee et al., 2003; Lee et al., 2004; De Bundel et al., 2009). In 1992, Wright and Harding identified binding sites for the hexapeptide Ang IV in several brain areas associated with cognition, motor and sensory physiological functions (Swanson et al., 1992), e.g., in hippocampus (Harding et al., 1992). The encouraging data obtained after administration of Ang IV in the experimental models and the impact of Ang IV on parameters anticipated to be linked to cognition promoted an interest in more detailed studies of the hexapeptide.

Excellent reviews on Ang IV and its contribution to cognition are available (Gard, 2008; Albiston et al., 2011; Ho and Nation, 2018; Jackson et al., 2018; Wright and Harding, 2019). Herein we summarize the efforts the last three decades to transform the hexapeptide into more drug-like peptidemimetics acting at its receptor(s). We discuss from a medicinal chemistry perspective, starting points in the design processes and the very different approaches applied. The incorporation of various unnatural amino acid residues into peptides was proven to be a very productive methodology. The iterative stepwise modifications of the parent ligand Ang IV is first presented.

## Linear Angiotensin IV Analogues and Incorporation of Unnatural Amino Acids

After systematic structure activity studies (SAR) of Ang IV analogues, involving glycine and D-amino acid scans in combination with displacement and incorporation of various alternative amino acid residues it became clear that the N-terminal Val-Tyr-Ile residues of the peptide ligands were important for high affinity to the specific binding site identified (Sardinia et al., 1993), named the AT4 receptor (de Gasparo et al., 1995). Furthermore, amino acid residues with hydrophobic side chains in the 1-position of the Ang IV analogues, e.g., a norleucine residue (cf. **2**) combined with an unsubstituted amino group in the N-terminal seemed optimal. On the contrary, alterations in the C-terminal seemed less critical for activity and for instance a deletion of the C-terminal phenylalanine residue of **1** had only a minor impact and the binding affinity was essentially maintained (Sardinia et al., 1993; Sardinia et al., 1994; Krishnan et al., 1999).

**Figure 2:**
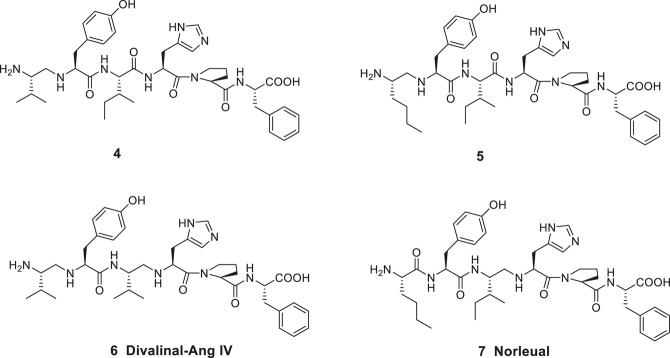
Linear peptides with high affinity to the angiotensin IV (AT4) receptor and that are less prone to proteolytic degradation.

Ang IV and related analogues are susceptible to proteolytic cleavage of the peptide bond between the Val^1^ and Tyr^2^ amino acid residues. Thus, a reduction of this N-terminal peptide bond rendered peptides significantly less prone to hydrolysis. Notably, most of the affinity to the binding site was still maintained. The Ang IV analogue peptide **4** and the Nle-Ang IV analogue **5** provide examples comprising two basic amino groups accessible for protonation in the N-terminal of the peptides and flanking hydrophobic 2-isopropyl and n-butyl side chains, respectively (Figure 2) (Sardinia et al., 1994). A reduced peptide bond between the amino acid residues Ile^3^ (or Val^3^) and His^4^ is the characteristic feature of divalinal-Ang IV **6** and norleual **7**, two pseudopeptides that both for a long time served as important research tools (Krebs et al., 1996; Kramár et al., 2001).

**Figure 3:**
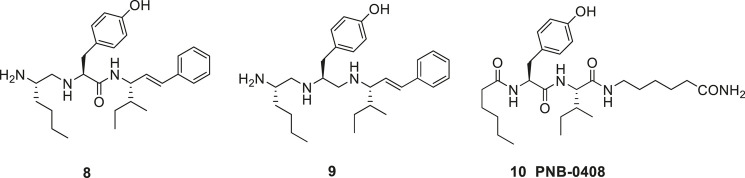
The low molecular weight high-affinity angiotensin IV (AT4) receptor binding analogues **8** and **9** incorporate one or two reduced peptide bonds. PNB-0408 (**10**), devoid of an N-terminal amino group crosses the blood-brain barrier, enhances cognition and is interfering with the hepatocyte growth factor/c-Met receptor system.

In the late 1990s Taisho Pharmaceuticals filed patent applications covering compounds that according to competitive experiments with radiolabeled [^125^I]Ang IV exhibited high affinity to hippocampus membranes from guinea pig (Kobori et al., 1997; Kobori et al., 1998). Kobori et al. (1997), Kobori et al. (1998) characterized the compounds as Ang IV receptor agonists and some of them, e.g., **8** with the straight four-carbon chain of the Nle residue at position one, exhibited very high affinity to the binding site (IC50 values < 1 nM) (Figure 3). The drug-like **8** encompasses a styrene moiety replacing His-Pro-Phe residues in the C-terminal of Ang IV. Furthermore, it was demonstrated that saturation of the *trans* styrene double bond of **8** was deleterious for activity. Reduction of the remaining peptide bond of **8** furnished **9**, a compound that still bind to the binding site although with 40 times lower affinity. From the series of compounds that were disclosed it appears that the binding site favors two amine functions in the N-terminal part of the ligands in order to achieve an efficient binding to the receptor (cf. **4**, **5**, **6**). Hence, a compound with an intact peptide bond between the Nle^1^ and Tyr^2^ residues but with a reduced peptide bond between the Tyr^2^ and Ile^3^ residues was essentially inactive (Kobori et al., 1997; Kobori et al., 1998).

In 2001, Albiston and Chai suggested that Ang IV exerts its procognitive actions by inhibition of a peptidase, insulin-regulated aminopeptidase (IRAP) (Albiston et al., 2001) and not through binding to a G-protein-coupled receptor as is the case of its major precursor angiotensin II (Ang II, Asp-Arg-Val-Tyr-Ile-His-Pro-Phe), that mediates its effects mainly through the Ang II receptor type 1 (AT1R) and the angiotensin receptor type 2 (AT2R). Thus, while the octapeptide Ang II exerts a powerful hypertensive effect and acts as a receptor agonist, it is degraded by proteolysis to a bioactive hexapeptide metabolite with a very different pharmacological profile that acts as an enzyme inhibitor. Notably, there are several other examples known demonstrating that small neuropeptides can be converted into fragments with significantly different biological effects, e.g., the nociceptive substance P is degraded to the antinociceptive substance P (1–7) (Fransson et al., 2008; Hallberg, 2015). Ang IV binds to both AT1R and AT2R but only at micromolar concentrations (Bosnyak et al., 2011; Hallberg et al., 2017). Furthermore, the IRAP inhibitors Ang IV (**1**) and LVV-hemorphin-7 (**3**) (Lee et al., 2003; Lew et al., 2003) inhibit also aminopeptidase N (AP-N, EC 3.4.11.2) activity (Garreau et al., 1998; Fruitier-Arnaudin et al., 2002) and LVV-hemorphin-7 (**3**), in addition, binds to the μ-opioid receptor (Nyberg et al., 1996).

IRAP (EC 3.4.11.3) is a single-spanning transmembrane zinc-metallopeptidase that belongs to the M1 family of aminopeptidases. The enzyme was cloned and characterized in adipocytes in vesicles containing the insulin-regulated glucose transporter GLUT4 (Keller et al., 1995; Keller, 2003), was identified as placental leucine aminopeptidase, oxytocinase, gp160 and vp165 and is a cystinyl aminopeptidase (Tsujimoto et al., 1992; Kandror and Pilch, 1994; Rogi et al., 1996; Rasmussen et al., 2000; De Bundel et al., 2008). IRAP is able to cleave the peptide bond between the N-terminal amino acid residues from several bioactive peptides *in vitro*, e.g., met-enkephalin and leu-enkephalin, dynorphin A, lysine-bradykinin, neurokinin A1, cholecystokinin-8, somatostatin, oxytocin and vasopressin (Lew et al., 2003) although the key substrates seem to be the macrocyclic oxytocin and arginine-vasopresin (Albiston et al., 2010a). Studies on the IRAP knockout (KO) mice suggest that vasopressin, oxytocin and somatostatin are physiologically important substrates (Wallis et al., 2007; De Bundel et al., 2015). Hence, the N-terminal peptide bonds of macrocyclic peptidic regulators of cognition, as vasopressin and oxytocin can be cleaved (Alescio-Lautier et al., 2000; Matsumoto et al., 2000; Matsumoto et al., 2001; Wallis et al., 2007). The aminopeptidase is expressed in areas of the brain associated with cognition (Fernando et al., 2005; Albiston et al., 2007; Albiston et al., 2011), and the observation that Ang IV improves memory and learning has attracted attention to the aminopeptidase IRAP as a potential macromolecular target for drugs for treatment of cognitive disorders (Wolfe, 2002; Gard, 2008; Wright and Harding, 2008; Hallberg, 2009). Moreover, the enzyme mediates a series of other important physiological activities that are related to immunology (Stratikos, 2014), e.g., antigen processing and trafficking of T-cells receptors and of glucose transporters (Waters et al., 1997; Bryant et al., 2002). The catalytic site of IRAP is located in the extracellular region and is highly homologous to two other important members of the M1 aminopeptidase family, endoplasmic reticulum aminopeptidase 1 (ERAP1) and 2 (ERAP2) (Evnouchidou et al., 2009). IRAP, ERAP1, and ERAP2 are the three so-called antigen processing aminopeptidases responsible for the processing of the N-terminus of antigenic peptides and the activity of the three enzymes constitutes an important regulatory node in antigen processing, *vide infra* (Stratikos, 2014).

It should be emphasized that macromolecular targets other than IRAP have been proposed to account for the positive physiological effects on cognition observed *in vivo* after administration of Ang IV and related analogues (Vanderheyden, 2009; Kawas et al., 2012; Benoist et al., 2014). The tyrosine kinase receptor c-Met that binds hepatocyte growth factor (HGF) and that is associated with memory and learning consolidation is one candidate (Wright et al., 2008; Yamamoto et al., 2010) being implicated in Alzheimer’s disease (AD) (Wright and Harding, 2011). Consequently, it is suggested that Ang IV exerts its action through the c-Met signaling pathway (Wright and Harding, 2019) and an overexpression HGF in the nervous system enhances learning and memory performance in mice (Kato et al., 2012). The Ang IV analogue Norleual **7** inhibits HGF-mediated effects at picomolar concentrations and blocks [^125^I]HGF binding to c-Met (Yamamoto et al., 2010). The drug-like PNB-0408 (N-hexanoyl-Tyr-Ile-N′-(5-carbamoylpentyl)amide **10**, synthesized in the laboratories of Wright and Harding is of particular interest and penetrates the blood-brain barrier and improves cognitive activity. (Figure 3) (Wright and Harding, 2008; Wright and Harding, 2009; McCoy, 2010). PNB-0408 (dihexa) with a primary amid function in the C-terminal attached via a lipophilic tether to the Ile residue is devoid of the N-terminal amino group found in most Ang IV analogues, *vide supra*. The compound **10**, derived from Nle-Ang IV **2** facilitates formation of new functional synaptic connections, facilitates LTP in hippocampal slices and amplifies memory consolidation in animal models of AD (Wright and Harding, 2015; Wright and Harding, 2019). Even though the brain hepatocyte growth factor/c-Met receptor system has attracted considerable attention (Wright and Harding, 2019), with regard to synthesis of small molecules and medicinal chemistry, it seems that most efforts so far have been devoted to identify new selective inhibitors of IRAP. To the best of our knowledge, no data on inhibition of IRAP by compounds **8** and **9** and related ligands from the Kobori laboratory nor by PNB-0408 (**10**) are yet available in literature.

After the pioneering work by Wright and Harding at Washington State University and by Kobori et al. (1997), Kobori et al. (1998). at Taisho Pharmaceuticals in Japan who identified low molecule strong binders to the Ang IV receptor in the 1990s, several groups have been engaged in the search for improved compounds interacting with the Ang IV receptor. The fundamental discovery by Albiston and Chai that IRAP is a molecular target for Ang IV and that IRAP seems to play an important physiological role and is associated with cognition encouraged focused research aimed at identifying new efficient IRAP inhibitors that could serve as pharmaceutical agents. Several complementary approaches were applied; 1) iterative modifications of the endogenous Ang IV, 2) virtual and substance library screening, and 3) direct design applying knowledge of the structure of IRAP. To make orally bioavailable IRAP inhibitors that reach the brain and that are metabolically relatively stable is a tremendous challenge.

Previously metal chelators (e.g., EDTA and phenantroline) were normally added in the experimental settings for determination of binding affinity. Although many of the ligands that in the 1990 ties had been identified as high-affinity binders inhibit IRAP (Lee et al., 2003; Lew et al., 2003), the ligands often demonstrated differences in potencies and rank orders in the IRAP assay in experiments where chelators were absent. It was postulated that the differences observed were attributed to the absence of zinc in the active site in presence of metal chelators (Lew et al., 2003; Demaegdt et al., 2004; Laeremans et al., 2005; Demaegdt et al., 2006). It became apparent that chelators must be omitted to obtain physiologically relevant data (Demaegdt et al., 2009).

**Figure 4:**
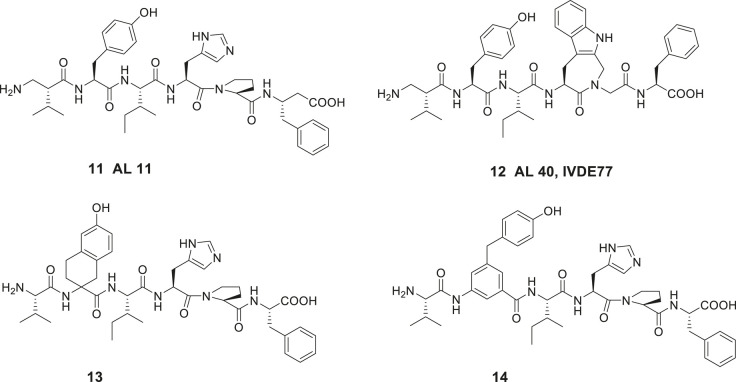
The high affinity and metabolically stable IRAP inhibitors **11** (AL-11) and **12** (AL-40) both comprising β amino acid residues. The compounds **13** and **14** where the Tyr^2^ residue is replaced are inactive as IRAP inhibitors.

Introduction of unnatural amino acids into peptide structures was demonstrated to provide a successful concept in the search for potent and metabolically stable inhibitors of IRAP. Hence, a *β*-homoamino acid scan of Ang IV performed at Vrije University in Brussels resulted in the identification of the metabolically stable derivative **11** (AL-11) with five-fold higher affinity to IRAP than Ang IV (Ki = 7.6 vs. 62 nM for Ang IV) and exhibiting a high selectivity for IRAP over AP-N (Figure 4) (Lukaszuk et al., 2008). Thus, insertion of methylene groups in both in the N- and C-terminals of Ang IV improved the inhibitors. The His^4^ and Pro^5^ residues of Ang IV were subsequently replaced by conformationally constrained residues and the highly selective and stable IRAP inhibitor **12** (AL-40, IVDE77) was identified, with >30-fold higher affinity (Ki = 1.7 nM) than Ang IV and improved both selectivity and metabolic stability (Lukaszuk et al., 2008; Lukaszuk et al., 2009; Nikolaou et al., 2013). In addition, the Belgium group assessed the roles of Tyr^2^, Pro^5^, and Phe^6^ in Ang IV by systematic incorporation of constrained residues. The replacements of Tyr^2^ by residues with restricted flexibility were deleterious for activity. For example, the compound **13** (racemic) is a very week IRAP inhibitor (Lukaszuk et al., 2011) and compound **14** with a 4-hydroxydiphenylmethane scaffold as a substitute for Tyr^2^ is essentially inactive (Figure 4) (Andersson et al., 2008). Thus, in summery the lipophilic character of the side chain in position **1** is essential and the proper orientation of the Tyr^2^ side chain in space is critical for activity and IRAP inhibition while various alternations and topography at the C-terminal are more acceptable.

## Macrocyclic Angiotensin IV Analogues

**Figure 5:**
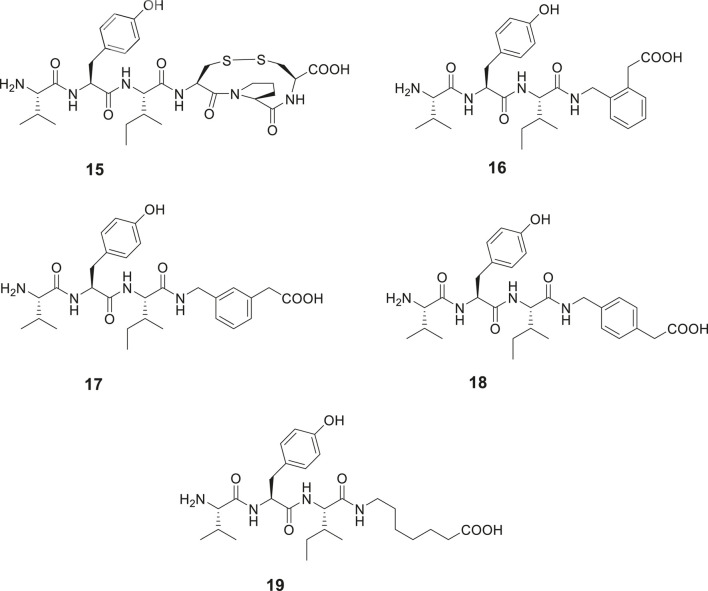
Modifications in the C-terminal. Compound **15** with an 11-membered macrocyclic ring adopting a γ-turn in the C-terminal binds better than Ang IV to IRAP and **16** with a scaffold serving as a γ-turn mimetic incorporated in the C-terminal is essentially as active as Ang IV. Compounds **17**, **18**, and **19** bind to IRAP but are more than five-fold less potent.

The capability of IRAP to degrade physiologically important cyclic peptides has inspired the development of macrocyclic analogues of Ang IV. Our group synthesized a series of macrocyclic peptides with the aim to determine bioactive conformations and better understand the mode of binding and structural requirements for efficient binding of Ang IV to IRAP (Axén et al., 2006; Axén et al., 2007; Andersson et al., 2010; Andersson et al., 2011). Steric constrains were introduced. Attempts to obtain high affinity binding inhibitors by cyclization in the C-terminal was productive as expected from previously obtained data. Hence, the macrocyclic disulphide **15** encompassing an 11-membered macrocycle was more potent as an IRAP inhibitor than the native Ang IV and exhibited a Ki value of 26 nM (Figure 5). Since the macrocyclic system of **15**, as deduced from modeling tends to adopt a γ-turn (Schmidt et al., 1997; Lindman et al., 2001), the entire C-terminal tripeptide fragment His-Pro-Phe was replaced by a 2-(aminomethyl)phenylacetic acid moiety anticipated to serve as a proper γ-turn mimic. The structurally simplified Ang IV peptidemimetic **16** was almost as active as Ang IV as an IRAP inhibitor while **17** was five-fold and **18** and the open chain **19** were 20-fold less active (Figure 5). Furthermore, compound **16** is degraded considerably more slowly in membrane preparations than Ang IV and stimulates proliferation of mouse neural stem cells at low concentrations, supporting a role of IRAP in neurogenesis (Axén et al., 2007).

**Figure 6:**
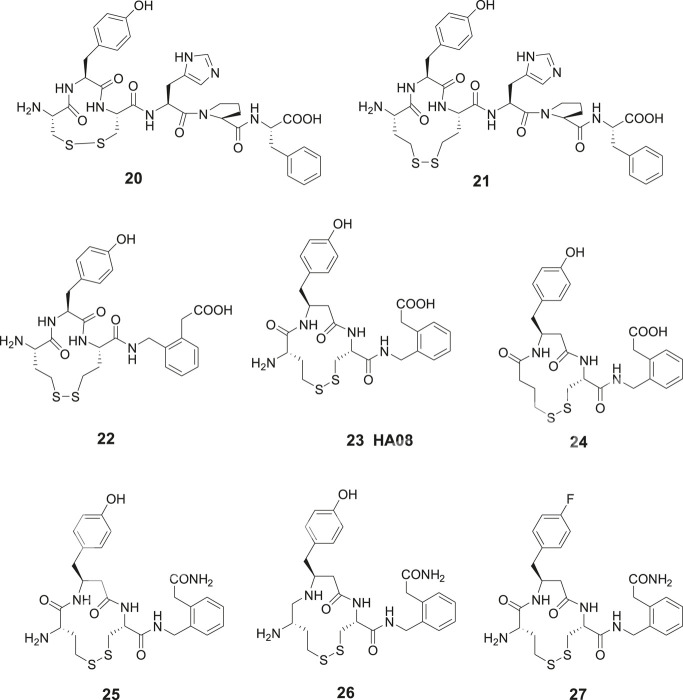
Macrocyclizations in the N-terminal. The Ang IV analogue **20** encompassing an 11-membered macrocycle is inactive while **21** with a 13-membered ring system inhibits IRAP efficiently. The selective compound **23** (HA08) is the most potent IRAP inhibitor in the series and deletion of the N-terminal amino group of **23** makes the ligand (**24**) inactive. The carboxamide **25** is two-fold and the fluoro compound **27** is 10-fold less potent than **23** as IRAP inhibitors. Compound **26** comprising a reduced peptide bond in the N-terminal is a weak IRAP inhibitor.

Contrary to the results obtained after modification in the C-terminal of Ang IV where essentially all compounds examined exhibited some activity, oxidative cyclization of the side chains of Cys^1^ and Cys^3^ and consequently creation of an 11-membered disulfide macrocycle in the N-terminal rendered the inactive compound **20** (Figure 6). However, a widening of the ring system was productive and the Ang IV analogue **21** with a 13-membered macrocycle exhibited a moderate IRAP inhibitory capacity. Compound **22**, a hybrid with the N-terminal from **21** and the C-terminal from **16** demonstrated a further enhanced potency (Ki = 23 nM) and a high selectivity for IRAP over AP-N (Andersson et al., 2010). Notably **16**, devoid of a macrocyclic ring system in the N-terminal is not selective and inhibits both IRAP and AP-N activity, suggesting that introduction of proper conformational constrains in the N-terminal by macrocyclization improves selectivity (Axén et al., 2007). As deduced from molecular modeling, **15** and Ang IV seem to adopt a *γ*-turn at the C-terminal when binding to IRAP, while in the case of **21**, a less well-defined turn conformation is adopted at the N-terminal. Further structural optimization delivered a large number of macrocyclic disulfides and among them **23** (HA08) encompassing a β3-homotyrosine residue in a 13-membered ring system and exhibiting the lowest Ki value in the series (Ki = 3.3 nM). HA08 is formed after an Hcy^1^/Cys^3^ side chain cyclization and is a competitive inhibitor and 20 times more potent than Ang IV and less prone to proteolytic degradation (Figure 6). The compound was designed to provide both high IRAP inhibitory capacity and an improved metabolic stability attributed to the introduction of an β-amino acid residue adjacent to the predicted scissile peptide bond. Enlargement of the ring system of **23**, by oxidatative cyclization of Hcy^1^/Hcy^3^ side chains rather than the Hcy^1^/Cys^3^ side chains of **23** resulted in a compound comprising a 14-membered macrocycle but with a somewhat lower affinity to IRAP (Ki = 5.1 nM), *vide infra*. Furthermore, one important characteristic of **23** (HA08) is its high 2,000-fold selectivity for IRAP over AP-N and high selectivity vs. the homologous enzymes ER aminopeptidase 1 (ERAP1) and ER aminopeptidase 2 (ERAP2). Replacement of the L-amino acids of **23** with D-amino acids were not productive (Andersson et al., 2010). Deletion of the N-terminal amino group of **23** had a dramatic effect. Hence, **24** is inactive as an IRAP inhibitor, cf. the potent cognitive enhancer **10** (PNB-0408, dihexa), derived from Ang IV, *vide supra*. Furthermore, removal of the methylene carboxy group at the C-terminal of **23** resulted in a less efficient IRAP inhibitor. Introduction of a primary carboxamide in the C-terminal (cf. PNB-0408, **10**) to replace the carboxy group of **23** provided **25** that inhibits IRAP but exhibits a two-fold lower activity (Figure 6). A reduction of the N-terminal peptide bond of **25**, with the ambition to achieve a compound even more resistant to proteolytic cleavage, provided **26** (Barlow et al., 2020). This maneuver was not fruitful and compound **26** displays a Ki value in the micromolar range in sharp contrast to what could be expected considering the results obtained after reduction of Ang IV or Nle-Ang IV, cf. **1** vs. **4** and **2** vs. **5** where the bioactivities were essentially retained after reduction of the carbonyl group of the peptide bond, *vide supra*. Moreover, in attempts to replace the hydroxyl group of the tyrosine moiety to avoid potential problems with phase II metabolism compound **27** was prepared (Barlow et al., 2020). This fluoro compound (**27**) is 10-fold less potent than **25** demonstrating that interactions with the hydroxyl group is important, although not critical for activity.

Crystal structures of IRAP were recently solved (Hermans et al., 2015; Mpakali et al., 2015). Based on analyses of available crystal structures a tentative model for **23** (HA08) binding to IRAP was created in 2016 by applying MD simulations and calculating associated binding free energies by the linear interaction energy (LIE) method. According to the model, the carbonyl group of the N-terminal peptide bond of **23** is coordinated to the Zn^2+^ ion, whereas the terminal amine is fixed by three glutamate carboxylates (Glu431, Glu487, and Glu295) (Diwakarla et al., 2016b). Regarding the affinity of the fluoro compound **27**, it was anticipated that the hydroxyl group of the tyrosine side chain of **25** makes a hydrogen-bond with Glu494 of IRAP, an interaction that is lost in the case of **27** according to the free energy perturbation (FEP) analysis (Jespers et al., 2019), explaining the reduction in binding affinity of compound **27** as compared to the analogous compound **25**. Moreover, it was postulated that the aromatic ring of the β3-homotyrosine side chain and the C-terminal phenyl ring interact and Phe550 in IRAP further stabilizes aromatic/hydrophobic packing (Barlow et al., 2020).

More recently, a crystal structure of IRAP with the macrocyclic peptide inhibitor **23** (HA08) was reported (Mpakali et al., 2020). After a comparison with the known IRAP structures and a combination with small angle X-ray scattering experiments it was proposed that IRAP is an open dimer in solution and assumes a more compact conformation upon HA08 binding. Thus, **23** (HA08) stabilizes the closed conformation of IRAP. Compound **23** is combining the structural elements from Ang IV and the physiological substrates vasopressin and oxytocin. The cleavage of the scissile bond of substrates as oxytocin and vasopressin is anticipated to take place after a nucleophilic attack by water assisted by the carboxylate function of Glu465 in the active site of IRAP. Importantly, the corresponding cleavage of the scissile bond of the 13-membered **23** (HA08) in the closed conformation of IRAP is less prone to occur since there is not enough space for motion of water molecules to interact with the carboxylate group of Glu465. In fact, no ordered water molecule is found in the crystal structure at that location. The structure reveals that the side-chain of the β-tyrosine amino acid residue is situated in the S1 pocket of the enzyme and that the disulfide bond abuts to the S1′ pocket. The phenylacetic acid group of HA08 was found in two different orientations; *a*) the carboxyl group makes electrostatic interactions with Arg439 and Arg929 of IRAP and *b*) phenyl ring of the phenylacetic acid group makes pi-stacking interactions with Tyr961. Moreover, it was suggested from inhibition data that **23** (HA08) acting through a competitive mechanism and Ang IV may occupy the same binding site and operate by the same mode of action. The structure of the IRAP (Mpakali et al., 2020) was found very similar to that reported previously with a phosphinic pseudotripeptide transition-state analogue binding to the enzyme, *vide infra* (Mpakali et al., 2017). It was concluded that HA08 binds to IRAP in a very similar conformation to what was proposed by the MD simulations (Diwakarla et al., 2016b; Barlow et al., 2020).

**Figure 7:**
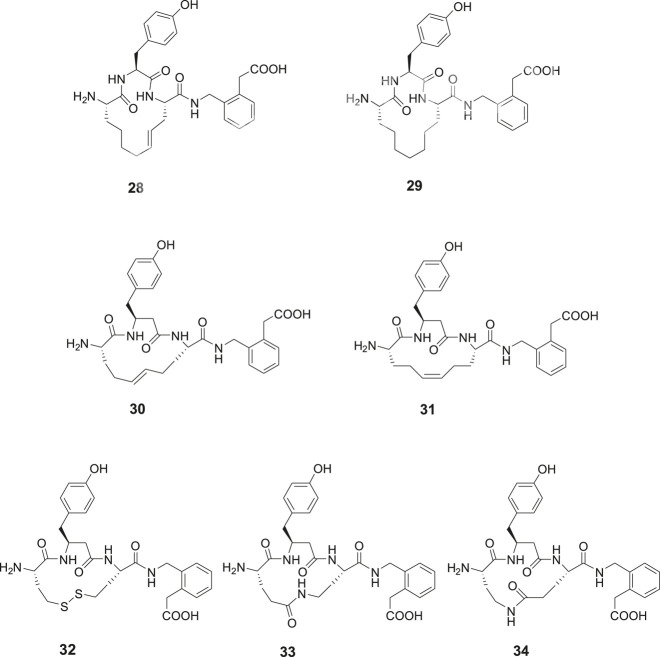
Macrocyclizations in the N-terminal. A comparison of the activity of a series of cyclized Ang IV analogues encompassing 14-membered ring systems. The macrocyclic **28** and **30**, made by metathesis reactions are the most potent IRAP inhibitors in the series and somewhat more potent than the disulfide analogue **32**. Compound **29** with a saturated carbon bridge and the cis isomer **31** are 10-fold less potent and the amides **33** and **34** more than 100-fold less potent than the most effective IRAP inhibitor compound **30**.

The metathesis reaction was applied to obtain a series of macrocyclic compounds, devoid of the reactive disulfide function and with the potential to become oral bioavailable and more metabolically stable (Driggers et al., 2008; Marsault and Peterson, 2011). Several carba analogues comprising a 13- or 14-membered heterocycle were synthesized and assessed (Andersson et al., 2011). Among those, the tyrosine derivative **28** exhibited a Ki value of 4.1 nM and the corresponding analogue with a saturated double bond, compound **29**, a Ki value of 25 nM, demonstrating that a proper constrain of flexibility of the ring system, alternatively an electron density in the center of the lipophilic bridge between amino acid residues in positions 1 and 3 is favorable for affinity to IRAP (Figure 7). Compound **30** encompassing a homo tyrosine in a 14-membered macrocycle and with a *trans* double bond provided the lowest Ki value in the series, 1.8 nM, while the corresponding tyrosine analogues with the 13-membered ring system exhibited a Ki value of 50 nM. Compound **31**, the *cis* isomer of **30**, demonstrated a lower affinity to the enzyme (Ki = 30.4 nM). The high activity of **30** prompted a synthesis of the corresponding disulfide **32** with a 14-membered ring system anticipated be able to adopt a similar “trans conformation” as **30**. A high activity was encountered, Ki = 5.1 nM, to be compared with **23** (HA08) with a Ki of 3.3 nM, the latter comprising a 13-membered macrocyclic system. Insertion of amide bonds at various locations in the carbon chain was not productive. The lactams **33** and **34** comprising 14-membered ring systems were most active in the series but nonetheless 100-fold less potent than the best disulfide and carba analogues (Barlow et al., 2020).

**Figure 8:**
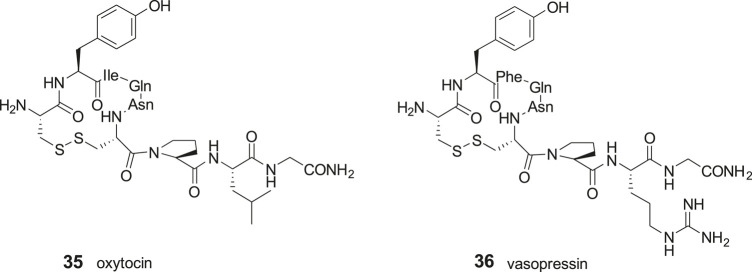
The macrocyclic disulfides oxytocin (**35**) and vasopressin (**36**) are IRAP substrates.

The disulfide and carba analogues examined demonstrated a relative high stability against proteolysis by metallopeptidases, despite the similarities in the N-terminal to oxytocin **35** and vasopressin **36** that both serve as substrates to IRAP (Alescio-Lautier et al., 2000; Matsumoto et al., 2000; Matsumoto et al., 2001; Wallis et al., 2007; Hermans et al., 2015). This is most likely attributed to the inability of the constrained 13- and 14-membered macrocycles to adopt the proper transition states required for cleavage of the N-terminal peptide bond (Andersson et al., 2011) (Figure 8).

To conclude, macrocyclizations by oxidative disulfide formations in the N-terminal of Ang IV analogues or alternatively displacement of the disulfide unite with a *trans* double bond can provide very potent inhibitors of IRAP, as exemplified with **23** (HA08) comprising a 13-membered ring system and **28**, **30**, and **32** comprising 14-membered ring systems. Macrocyclization can convey drug-like properties of larger molecules (Driggers et al., 2008; Brandt et al., 2010; Marsault and Peterson, 2011; Mallinson and Collins, 2012; Giordanetto and Kihlberg, 2014) that includes improved membrane permeability compared to acyclic matched pairs and oral bioavailability but also often improved affinity for the target, improved selectivity and reduced metabolism (Hess et al., 2008; Bogdan et al., 2011; Yap et al., 2016).

The macrocycle **23** was selected as a proper research tool for more detailed studies since the molecule is convenient to prepare and due to its structural similarity to the IRAP substrates oxytocin and vasopressin in its N-terminal. A decline in the dendritic spine density (DSD) is an early characteristic feature of many neurodegenerative diseases (Fiala et al., 2002; Bourne and Harris, 2008; van Spronsen and Hoogenraad, 2010; Penzes et al., 2011) and drugs able to enhance DSD are suggested as potential candidates for future treatment of memory disorders (Lynch et al., 2008). Dendritic spines, small protrusions from the dendrites that acts as contacts with neighboring axons and contain all of the molecular machinery required for synaptic plasticity and storage of memories. Hence, the procognitive activity of a molecule *in vivo* correlates well with its *in vitro* ability to alter dendritic spine structure and DSD (Moser et al., 1994; O’Malley et al., 1998; O’Malley et al., 2000; Benoist et al., 2011; Fu et al., 2012; Lai et al., 2012; McCoy et al., 2013). The specific loss of stubby/mushroom spines, which morphologically are characterized by a larger spine head and functionally have stronger synapses (Nusser et al., 1998; Matsuzaki et al., 2001; Murthy et al., 2001) is assumed to have a more pronounced impact on cognitive decline (Kasai et al., 2003). The competitive IRAP inhibitor **23** (HA08) enhances the numbers of dendritic spines in hippocampal cell cultures and HA08 treatment resulted in an increasing number of stubby and mushroom-like spines, a morphology typically associated with mature spines, which are believed to have strengthened synaptic connectivity. The dendritic spines were also vesicular glutamate transporter 1 (vGLUT1) positive, indicating that spines were receptive to glutamatergic signaling. Notably, in contrast to **23** (HA08), a structurally very similar epimer (HA09) which differs only with regard to one stereogenic center and exhibiting a 100-fold lower affinity to IRAP did not affect spine morphology, indicating a correlation between the capacity to inhibit IRAP and a positive impact on DSD and spine morphology. HA09 comprises a Cys^3^ residue with *R* rather than *S*-configuration. The effect of **23** (HA08) was similar to that of brain-derived neurotrophic factor (BDNF) that is a known inducer of spine development (Diwakarla et al., 2016b).

## Small Molecule Insulin-Regulated Aminopeptidase Inhibitors From *In Silico* Screening

**Figure 9:**
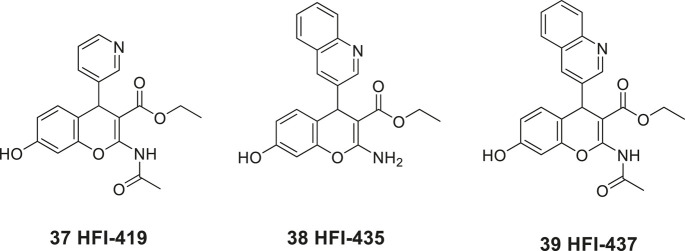
The first drug-like small molecule IRAP inhibitors reported. The benzopyran derivatives **37**, **38**, and **39** were discovered after *in silico* screening.

In 2008, Siew Chai and her group in Australia disclosed the first generation of drug-like small molecular weight inhibitors of IRAP (Albiston et al., 2008). An *in silico* screening of a homology model of IRAP based on the crystal structure of LTA4H (Leukotriene-A4 hydrolase) was applied (Thunnissen et al., 2001). The virtual screening of a library of approximately two million compounds resulted in a lead series of inhibitors encompassing a benzopyran scaffold. Among the more potent compounds were the racemic pyridine derivative **37** (HFI-419), and the quinoline derivatives **38** (HFI-435) and **39** (HFI-437) exhibiting *K*_*i*_ values of 420, 360, and 20 nM, respectively (Figure 9). The benzopyran-based IRAP inhibitor **37**, with high selectivity vs. aminopeptidases such APN, ERAP1, ERAP2, and LTA4H, exerted a cognitive-enhancing effect in rodents after *icv* administration similar to that of Ang IV **1** and LVV-H7 **3** (Albiston et al., 2008; De Bundel et al., 2009).

As deduced from computational docking the *S*-isomer is the preferred binding mode of the inhibitors although alternative binding conformations were suggested (Albiston et al., 2010b) However, after determination of the crystal structure of IRAP, computational docking of, e.g., **37**, **38**, and **39** into the IRAP structure demonstrated that these inhibitors all bind in the same orientation relative to the active site (Hermans et al., 2015), in contrast to conclusions drawn in previous modeling studies. Moreover, a rationale for the unique specificity of IRAP to process endogenous macrocyclic peptides such as oxytocin (**35**) and vasopressin (**36**) was presented (Hermans et al., 2015). Structure–activity relationship of a large series of benzopyran analogues has been established and the structural elements most important for binding have been determined. The lead candidate **37** (HFI-419) exhibits brain exposure following intravenous administration in rats but was found to be rapidly degraded to the corresponding deacetylated and less active IRAP inhibitor (Mountford et al., 2014).

An increase in the activity of matrix-metalloproteases (MMPs) or an increase in neuronal glucose uptake are two likely mechanisms by which inhibition of IRAP can provide an enhancement of memory. Modulation of either of the two systems is known to improve memory and learning as well as affect DSD. The benzopyran **37** (HFI-419) enhances spatial working memory in rats and the inhibition of IRAP by **37** increased DSD prior to peak dendritic growth in hippocampal neurons (Seyer et al., 2020). Moreover, it was concluded that this enhancement was likely to be driven by GLUT_4_-mediated changes to DSD. In particular, the inhibition of IRAP led to an enhancement of the proportion of mushroom/stubby-like spines. Furthermore, the spines were estimated to be functional based on their expression of the pre-synaptic markers vesicular glutamate transporter 1 and synapsin. The spine formation was inhibited in the case when the GLUT_4_-mediated glucose uptake was blocked. Thus, these results strongly suggest that IRAP inhibitors may facilitate memory by increasing hippocampal DSD via a GLUT_4_-mediated mechanism (Seyer et al., 2020).

## Small Molecule Insulin-Regulated Aminopeptidase Inhibitors From Substance Library Screening

**Figure 10:**
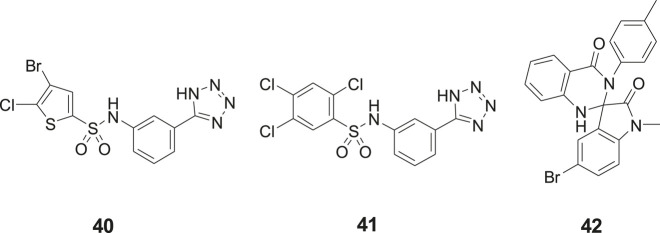
The sulfonamides **40** and **41** and the spiro-oxindole dihydroquinazolinone **42** were identified after screening a substance library of 10,500 low-molecular-weight compounds.

Our laboratory screened a substance library screen of 10 500 low-molecular-weight compounds in an enzyme inhibition assay with IRAP originating from Chinese hamster ovary (CHO). Three structurally different classes of compounds considered to be of particular interest as starting points for the development of small-molecule IRAP inhibitors were identified. The arylsulfonamide **40** with no structural similarities to Ang IV was one of them (Engen et al., 2016) (Figure 10). Compound **40** comprises a tetrazole ring in the meta position of an aromatic ring of an arylsulfonamide which is the characteristic feature of the sulfonamide class of compounds. Subsequently a large series of tetrazole derivatives were prepared and examined (Borhade et al., 2014) and a few of them were studied in detail. For example **41**, that is a competitive inhibitor of IRAP and exhibits an IC50 of 540 nM in a recombinant human IRAP assay. This sulfonamide (**41**) demonstrates a high metabolic stability and alters dendritic spine morphology and increases spine density in primary cultures of hippocampal neurons (Diwakarla et al., 2016a; Vanga et al., 2018). Molecular dynamics simulations and binding affinity estimations with the linear interaction energy method were performed for a large series of the arylsulfonamides. The significant agreement with experimental affinities suggested one of several tentatively proposed binding modes. Thus, the side chain of Arg439 of IRAP was estimated to interact with the tetrazole ring of the inhibitors while one of the oxygens of the sulfonamide function binds to the zinc ion of the enzyme. The NH of the sulfonamide binds to Glu 431 och Glu 295 via a water molecule bridge. This proposal was supported by the essentially perfect correlation for binding affinity differences between the selected pair of compounds obtained by rigorous free energy perturbation calculations by Gutierrez-de-Teran’s group (Vanga et al., 2018).

In addition to the sulfonamides, e.g., **40** and **41**, the spiro-oxindole dihydroquinazolinone derivative **42** was identified in the screening campaign and subsequently a large series of related analogues were made, e.g., by applying rapid MW-assisted reactions and thereafter examined as IRAP inhibitors in bioassays (Engen et al., 2020) (Figure 10). Compounds that were selective toward the closely related APN and that exhibited sub-μM affinity were identified. Enantiomers were separated and according to computational modeling the inhibitory capacity of the compounds were attributed to the *S*-configuration of the spiro-oxindole dihydroquinazolinones. The derivatives with corresponding *R*-configuration were postulated to be essentially inactive in all cases. Notably, the proposed binding mode is compatible with the simultaneous binding of the substrate L-Leu-*p*NA, in agreement with the uncompetitive IRAP inhibition determined for a representative compound. Unfortunately, the compounds suffer from poor *in vitro* metabolic stability (Engen et al., 2020).

## Direct Design From the Structure of Insulin-Regulated Aminopeptidase

**Figure 11:**
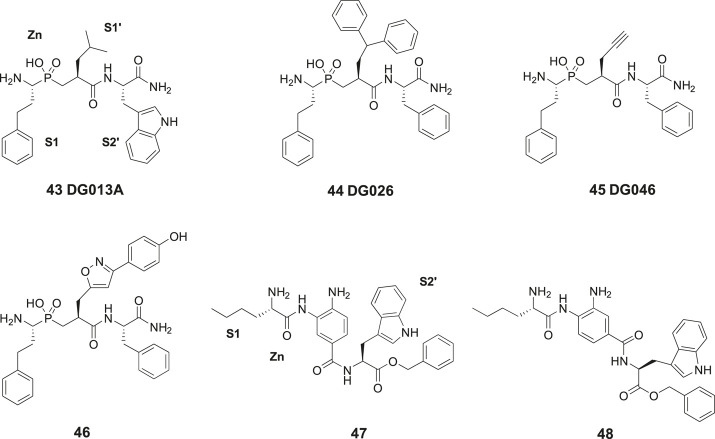
The phosphinic pseudotripeptide **43** (DG013A) is a potent IRAP inhibitor but is not selective and is equally effective inhibiting both ERAP1 and ERAP2. Compound **44** (DG026) comprising a bulky P1ʹ substituent is more IRAP selective than **43**. Compounds **45** (DG046) and **46** that are further modified in the P1ʹ position are the most potent IRAP inhibitors in the series and approximately 10-fold selective vs. ERAP1 and ERAP2. The IRAP inhibitors **47** and **48** encompassing a 3,4-diaminobenzoic acid (DABA) scaffold are significantly less efficient than **43–46** as IRAP inhibitors.

In 2013, laboratories in Greece and Stratikos’ and Georgiadis’ groups reported that the phosphinic pseudotripeptide **43** (DG013A) is a potent inhibitor of IRAP with an IC50 of 57 nM (Figure 11). In contrast to **23** (HA08), the pseudotripeptide **43** was not selective and the related ERAP1 and ERAP2 were inhibited as well showing IC50 values of 48 and 80 nM, respectively (Zervoudi et al., 2013). As deduced from an X-ray crystallographic analysis of **43** in complex with ERAP2 it was concluded that the three first specificity pockets of the enzyme was of particular importance for binding to the enzyme (Zervoudi et al., 2013). The two oxygen atoms of the hydroxyphosphinyl group of **43** are interacting with the zinc ion whilst hydrogen bonds with Glu371 and Tyr455 essential for catalysis are created. Phosphinic peptides often share structural similarities with the transition-state of peptide substrate upon hydrolysis (Georgiadis and Dive, 2015).

The phosphinic group is, as compared to, e.g., thiols and hydroxamic acids more weakly binding to zinc ions in metalloproteases and a significantly improved selectivity could be achieved by systematic variations of the side chains at P1′ and P2′ positions of **43**. Thus, the affinity to IRAP was improved after an enlargement of the P1ʹ as exemplified by **44** (DG026) exhibiting an IC50 of 32 nM while the affinity to ERAP1 dropped 100-fold. The crystal structure of IRAP in complex with **44** reveals that the enzyme undergoes structural reconfiguration that allows the accommodation of bulky side chains of the inhibitor. A closed conformational state of IRAP is created that is believed to be induced upon ligand binding. A hollow structure is formed excluding access of external solvent to the catalytic center (Mpakali et al., 2017). Notably, a propargyl group at P1′ or alternatively an extended P1′ side chain resulted both in very potent and fairly selective IRAP inhibitors, **45** (DG046) with IC50 values of 2 nM and **46** with a IC50 value of 4 nM (Figure 11). Thus, the potent IRAP inhibitor **45** demonstrated for ERAP1 an IC50 of 43 nM and for ERAP2 an IC50 value of 37 nM. The corresponding IC50 values for the oxazole derivative **46** with the extended P1ʹ side chain were 35 and 57 nM, respectively (Kokkala et al., 2016). Recently, a high-resolution crystal structure of phosphinic pseudopeptide inhibitor **45** (DG046) in the closed-conformation of ERAP1 was disclosed (Giastas et al., 2019b) and a mechanism for antigen peptide selection by ERAP1 presented (Giastas et al., 2019a). A review on inhibitors ERAP1 and ERAP2 was recently published (Georgiadis et al., 2019).

In 2015, Papakyriakou et al. reported a large series of inhibitors of ERAP1, ERAP2, and IRAP. This set of compounds comprises a 3,4-diaminobenzoic acid (DABA) scaffold (Papakyriakou et al., 2015). Compound **47** exhibited the best IRAP inhibitory capacity (IC50 = 105 nM) and demonstrated an almost 10-fold selectivity vs. ERAP1 (IC50 = 900 nM) and 15-fold improved selectivity vs. ERAP2 (IC50 = 1,600 nM) (Figure 11). L-Nle was preferred in the N-terminal for optimal inhibition and zinc coordination to the oxygen of the benzamide function is estimated to allow the lipophilic P1 substituent to be easily accommodated in the S1 pocket. In such conformation, the terminal amine group can form salt bridge interactions with two conserved glutamates, Glu295 and Glu431. Furthermore, the free aniline group could form a hydrogen bond with the catalytic E465 residue of IRAP. Deprotection of **47** to furnish an inhibitor with a carboxylate in the C-terminal exhibited a somewhat lower binding affinity for IRAP (IC50 = 296 nM). L-Val-OBn rather than L-Trp-OBn in the C-terminal led to a ten times less potent inhibitor of IRAP. The IRAP inhibitor **48** was the best inhibitor in a second series of bioisosteric diaminobenzoic acid inhibitors but was both less potent and selective as compared **47** (Papakyriakou et al., 2015).

Most small molecule binders of the oxytocinase subfamily of M1 aminopeptidases, e.g., the phosphinic pseudotripeptides **43–46** address the active site and establish strong interactions with the catalytic zinc ion. Highly potent enzyme inhibitors can be achieved but the selectivity obtained is often insufficient. Vourloumis’ group explored a large series of weaker zinc binding groups as potential alternatives to, e.g., the zinc binding amide function found in the 3,4-diaminobenzoic acid derivatives **47** and **48** (Tsoukalidou et al., 2019). Functionalized pyridinone- and pyridinethione-scaffolds, nicotinic-, isonicotinic-, aminobenzoic- and hydrazinobenzoic acids were prepared and examined as bioisosters, but no significant improvement of affinity was encountered by this maneuver. It was concluded that the potency of the compounds in the oxytocinase subfamily is mainly attributed to the occupation of the active site specificity pockets and their orientation in the enzymes (Tsoukalidou et al., 2019).

## Conclusion

A series linear Ang IV analogues demonstrating improved metabolic stability and very high affinity to IRAP have been reported, e.g., the potent hexapeptides **11** (AL-11) and **12** (IVDE77) with Ki values of 7.6 and 1.7 nM, respectively, data to be compared with the Ki value of Ang IV of 62 nM in the same binding assay. These pseudopeptides that have been studied in some detail are encompassing β-amino acid residues and **12**, in addition a conformationally constrained residue close to the C-terminal of the peptide. Furthermore, various macrocyclizations of Ang IV and subsequent simplifications of the structures delivered a series of selective high affinity ligands mimicking the substrates oxytocin and vasopressin in the N-terminal. For example, the disulfide **23** (HA08) that comprises a 13-membered ring system, a γ-turn mimetic in the C-terminal and a β-amino acid residue in the N-terminal ring system exhibits a Ki of 3.3 nM. The density of dendritic spines is closely associated with memory enhancement and **23** was found to increase the density of spines in the hippocampus significantly. To summarize, although very potent IRAP inhibitors have been discovered after systematic alterations starting from Ang IV none of the compounds are expected to reach the brain, at least not after oral administration.

Furthermore, ligands derived from the hexapeptide Ang IV but that are not proven to act as IRAP inhibitors have been reported. Thus, according to patent literature the drug-like **8** and **9** exhibit a very high affinity to the Ang IV binding site named the AT4 receptor. Moreover **10** (PNB-0408), a low-molecular weight molecule lacking the N-terminal amino group found in Ang IV analogues enhances cognition and is reported to crosses the blood-brain barrier and subsequently exert its action by interacting with the hepatocyte growth factor/c-Met receptor system. The compound has been studied in detail and promising pharmacological data have been reported. We believe that the positive effects of **10** on cognition are not attributed to inhibition of IRAP.

The HFI series of compounds, e.g., **37** (HFI 419) identified in 2008 after the virtual screening are much more drug-like than the hexapeptides **11** (AL-11), **12** (IVDE77), and the potent macrocyclic Ang IV analogue **23** (HA08) and can hopefully after further systematic optimization be converted into bioactive small molecules with capacity to cross the blood-brain barrier. However, while HFI 419 exerts a proven cognitive-enhancing effect in rodents after *icv* administration similar to that of Ang IV **1** and LVV-H7 **3**, the Ki value of 420 nM seems not optimal. The high throughput screening campaign of the substance library provided several hit compounds one of those the drug-like aryl sulfonamide **40** that was subsequently optimized into **41** that is a metabolically stable and reversible IRAP inhibitor. The sulfonamide **41**, like **23** (HA08) and **37** (HFI 419) increases spine density in primary cultures of hippocampal neurons but is unfortunately a too poor inhibitor of IRAP and is demonstrating a Ki value of 540 nM. Similarly, compounds from the spiro-oxindole dihydroquinazolinone series identified in the screening, e.g., **42** are weak inhibitors and besides that not stable in *in vitro* assays.

The phosphinic pseudotripeptide series of compounds designed from structural and mechanistic knowledge of IRAP and the related ERAP1 and ERAP2 are promising and some of them exert powerful IRAP inhibitory effects. Thus, pseudotripeptides **45** and **46** exhibit Ki values of 2 and 4 nM, respectively and an approximately 10-fold selectivity vs. ERAP1 and ERAP2. This class of inhibitors holds promise for the future. The 3,4-diaminobenzoic acid series of compounds, e.g., **47** with a Ki value of 105 nM are less efficient IRAP inhibitors.

In summary, both direct design relying on mechanistic knowledge and available structural data from the oxytocinase subfamily of M1 aminopeptidases as well as iterative manipulations of the parent peptide Ang IV, involving insertion of unnatural amino acids as β-amino acids, macrocyclizations and various structural simplifications have provided highly potent selective Ang IV peptidemimetics inhibiting IRAP. Nevertheless, no inhibitor has yet reached phase I clinical trials and further efforts are needed to circumvent all obstacles related to absorption, metabolism and various issues on pharmacokinetics in order to eventually achieve orally bioavailable drug candidates acting as cognitive enhancers *in vivo*.

## Author Contributions

MH and ML contributed equally in the preparation of the manuscript.

## Funding

We thank the Kjell and Märta Beijer Foundation, the Swedish Brain Foundation and King Gustaf V and Queen Victoria’s Foundation of Freemasons for economic support.

## Conflict of Interest

The authors declare that the research was conducted in the absence of any commercial or financial relationships that could be construed as a potential conflict of interest.The handling editor declared a past co-authorship with the authors.
